# Augmenting authenticity for non-invasive in vivo detection of random blood glucose with photoacoustic spectroscopy using Kernel-based ridge regression

**DOI:** 10.1038/s41598-024-53691-z

**Published:** 2024-04-09

**Authors:** P. N. S. B. S. V. Prasad V, Ali Hussain Syed, Mudigonda Himansh, Biswabandhu Jana, Pranab Mandal, Pradyut Kumar Sanki

**Affiliations:** 1 Department of Electronics and Communication Engineering, SRM University -AP, Neerukonda, 522240 India; 2 Department of Computer Science and Engineering, SRM University -AP, Neerukonda, 522240 India; 3 Department of Electrical and Electronics Engineering, ABV-IIITM Gwalior, Gwalior, MP 474015 India; 4 Department of Physics, SRM University -AP, Neerukonda, 522240 India

**Keywords:** Biomedical engineering, Diagnosis, Sensors and probes, Optical spectroscopy, Diode lasers

## Abstract

Photoacoustic Spectroscopy (PAS) is a potential method for the noninvasive detection of blood glucose. However random blood glucose testing can help to diagnose diabetes at an early stage and is crucial for managing and preventing complications with diabetes. In order to improve the diagnosis, control, and treatment of Diabetes Mellitus, an appropriate approach of noninvasive random blood glucose is required for glucose monitoring. A polynomial kernel-based ridge regression is proposed in this paper to detect random blood glucose accurately using PAS. Additionally, we explored the impact of the biological parameter BMI on the regulation of blood glucose, as it serves as the primary source of energy for the body’s cells. The kernel function plays a pivotal role in kernel ridge regression as it enables the algorithm to capture intricate non-linear associations between input and output variables. Using a Pulsed Laser source with a wavelength of 905 nm, a noninvasive portable device has been developed to collect the Photoacoustic (PA) signal from a finger. A collection of 105 individual random blood glucose samples was obtained and their accuracy was assessed using three metrics: Root Mean Square Error (RMSE), Mean Absolute Difference (MAD), and Mean Absolute Relative Difference (MARD). The respective values for these metrics were found to be 10.94 (mg/dl), 10.15 (mg/dl), and 8.86%. The performance of the readings was evaluated through Clarke Error Grid Analysis and Bland Altman Plot, demonstrating that the obtained readings outperformed the previously reported state-of-the-art approaches. To conclude the proposed IoT-based PAS random blood glucose monitoring system using kernel-based ridge regression is reported for the first time with more accuracy.

## Introduction

Diabetes Mellitus (DM) is a chronic condition characterized by either enough production of insulin or stop completely the production of insulin secretion by the pancreas. Prolonged high-value of blood sugar levels can have long-term effects including disorders like Neurological abnormalities, Heart attacks, and Renal failure. The World Health Organisation (WHO)^1^ and the International Diabetic Federation Association (IDFA)^2^ estimates that there are currently 450 million diabetics worldwide, and it will be around 700 million by the year 2045. Despite the fact that there is no cure for diabetes, it may be regulated by frequent monitoring of blood glucose. The progression and development of diabetes and associated disorders can be prevented by tight control of blood glucose levels. The currently available methods, however, rely on enzyme reactions and necessitate intrusive blood collection by painfully lancing the fingertip. The negative impact of this procedure on diabetic patients is twofold - it not only dissuades them from checking their glucose levels as frequently as recommended by medical professionals but also exposes them to the heightened risk of infection, potentially leading to severe consequences for their overall well-being. Recently Medtronic, Dexcom, and Abbott have introduced devices that enable continuous monitoring of blood glucose fluctuations in the interstitial fluid. However, these devices are also invasive, and the cost of the device is relatively high due to the regular replacement of the sensor in a periodic intervals. Numerous attempts have been made to develop noninvasive glucose detection methods that are equivalent to the currently available invasive methods in order to address these problems. The methods are divided into two categories: (i). Non-optical and (ii) Optical methods. The Non-optical methods are Iontophoresis^3^, Electrical Impedance^4^, Microwave based measurements^5^ and the Optical methods include Raman Spectroscopy^6–8^, Optical Coherence Tomography (OCT)^9,10^, Diffuse Reflection Spectrocopy (DRS)^11^, Mid Infra Red Spectrsocpy (MIR)^12^, Near Infra Red Spectroscopy (NIR)^13^ and Photoacoustic Spectrsocopy (PAS)^14^. Optical tissue window^15^ is considered for the optical methods where interference^16,17^ factors due to tissue layers and blood analytes are less effective. Among all the spectroscopy techniques, PAS^18–23^ has shown more potential for the noninvasive in vivo detection of glucose. This method, which relies on the Photoacoustic (PA) effect, causes acoustic waves to be generated in a sample after it has been excited by a monochromatic light source, such as a pulsed laser^24^. The sample absorbs optical energy and converts it into heat, resulting in a localized temperature rise and expansion followed by a contraction due to the off state of the laser. The periodic excitation of the laser leads to the generation of a pressure wave that travels from the irradiated region to the sample surface. PAS^19,20,23^ closely resembles conventional optical spectra and offers a noninvasive method to estimate blood glucose concentration by identifying specific excitation wavelengths that generate PA waves in tissue. PAS achieves high sensitivity and can detect even subtle variations in sample properties. PAS is capable of measuring absorption coefficients as low as 10$^{-7}\, cm^{-1}$, which makes a precise and effective technique for monitoring changes in glucose concentration. A pulsed laser light of wavelength 905 nm which absorbs more glucose molecules near the tissue has been used for the PA measurement in the NIR spectrum by which a promising result is generated. Dual-wavelength pulsed lasers have been used in implementing the PAS for the detection of blood glucose as reported by Pai et al.^19,21^ and they consider the change in the amplitude of the PA signal to represent the glucose value with a Gaussian kernel-based regression and the results are reported for in vitro testing by using the dual-wavelength lasers. Sim et al.^20^ utilized PAS and incorporated the Partial Least Square Regression Algorithm (PLSR) to detect glucose. They considered spatial information by scanning the finger and specifically targeting both secreting and non-secreting pores of the skin. The results might be impacted since the skin’s spatial information varies and the interpretation of periodic testing takes more time. Srichan et al.^25^ considered the multiple photonic near-infrared band sensor augmented with Personalized Medical Features (PMF) like SPO$_2$, Blood Pressure, Height, Weight, Age, Sex, and sensor values have been considered as the inputs to the shallow dense neural network implementation for blood glucose estimation. Although all the mentioned PMF might not create an impact on the detection of glucose molecules. So far the aforementioned PAS methods implemented dual-wavelength lasers or scanned finger images which are costly and take more time to produce the test results. Multivariate calibration algorithms provide robustness against abrupt fluctuations in a few feature values^26^. However, a careful balance must be maintained between model complexity and estimation error, as complex models risk overfitting to training data while simple models may lack generalizability. Additionally, the calibration model should be adaptable to incorporate additional inputs and signal features as necessary. The polynomial kernel-based ridge regression model^27–29^ is suitable for multivariate calibration and abruptly it will identify the changes in the parametric ratios. However, we identified only amplitude values from PA measurements are not sufficient for the noninvasive in vivo detection of random blood glucose. Here we incorporated the amplitude values from PA measurement and physically measured Body Mass Index (BMI) values of a person in the calibration algorithm. It is traced out that the BMI^30–33^ posses a strong influencing factor for the detection of random blood glucose compared with other medical features of a person. BMI is a measure of a person’s body fat based on their height and weight and it is an important factor essentially related to the use and regulation of glucose which is the main source of energy for the body’s cells. PAS is a potential method for the noninvasive detection of blood glucose. However, random blood glucose testing can help to diagnose diabetes at an early stage and is crucial for managing and preventing complications with diabetes. To improve the diagnosis, control, and treatment of DM, a portable cost-effective improvised IoT-based PAS setup has been proposed first time for self-monitoring of blood glucose in this work using the ThingSpeak Cloud platform. In addition, a polynomial kernel-based ridge regression is proposed in this paper to detect random blood glucose more accurately using PAS. The kernel function plays a pivotal role in kernel ridge regression as it enables the algorithm to capture intricate non-linear associations between input and output variables for better accuracy.

## Background

### Working principle of PAS

According to the Beers-Lambert law^34^, the intensity of incident optical energy, ${E_{a}}$ falls down exponentially as it penetrates through the sample with a depth of z. The glucose present in the sample with absorption coefficient $\alpha$ yields the optical absorption. The temperature of the medium rises as a result of the absorbed energy, creating volumetric expansion and it will be contracted during the absence of ${E_{a}}$. A periodic exposure of optical energy causes continuous stress and strain in the medium which creates a pressure wave ${p_{o}(z)}$^35^ and is given as

1 $$\begin{aligned} {p_{o}(z)}= \frac{\alpha \beta B}{\rho C_{p}} E_{o} e^{-\alpha Z} \end{aligned}$$

where $\beta$ is the thermal expansion coefficient of the sample and B is the isothermal bulk modulus, $\rho$ is the density and $C_{p}$is the specific heat. By utilizing the initial pressure distribution, ${p_{o}(t)}$, and the temporal profile of the optical excitation pulse g(t), the PA pressure^36,37^ generated in a sample can be determined and is given as.

2 $$\begin{aligned} P_{pa} = \frac{1}{\pi R^{2}}\left[ \frac{\alpha \beta \upsilon ^2}{C_{p}} \right] E_{a}. \end{aligned}$$

where,

$\alpha$ = Optical absorption coefficient.

$\beta$ = Thermal expansion coefficient.

υ = Acoustic velocity in the sample.

R = Radius of the PA source generated. The change in the glucose concentration $g_{ch}$ influences the physical properties of the sample and it is proportional to $\alpha$, $\beta$, $\upsilon$, & $C_{P}^{-1}$. The combined change in sample properties leads to an enhanced PA response, which correlates with the increase in sample glucose concentration. The amplitude of the PA signal is varied according to the change in the glucose levels.

## Methodology

### Proposed method for glucose detection

**Figure 1:**
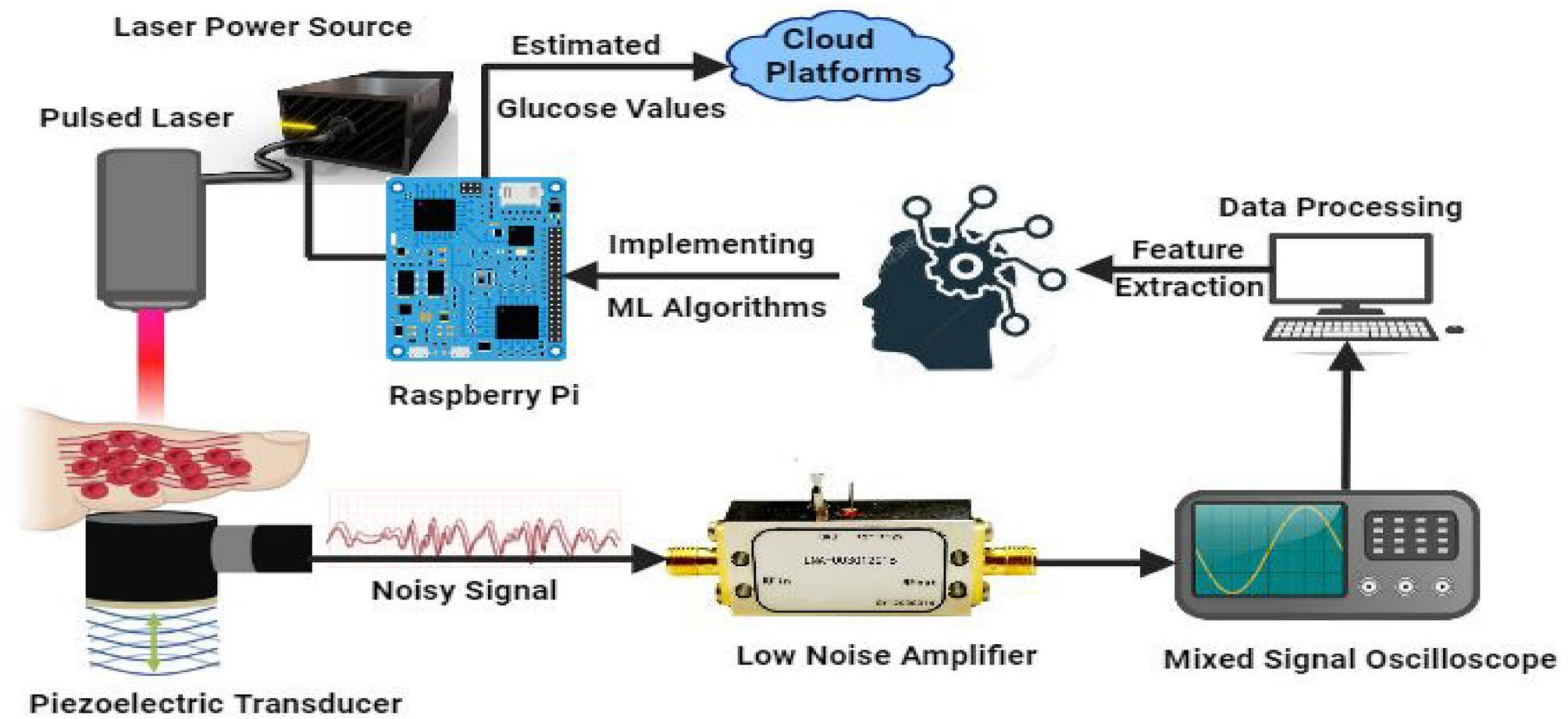
Block diagram of the proposed approach for determining blood sugar value using PA signal.

The block diagram of the proposed method is depicted in Fig. 1 showing a laser light with a peak power of 100W projected onto the finger with a wavelength 905 nm, Pulse Repeating Frequency (PRF) of 100 Hz, and a pulse width of 100ns. The laser light causes localized volumetric expansion, leading to the generation of a pressure wave. This pressure wave is detected by a piezoelectric transducer, which converts it into an output voltage. The resulting voltage is utilized for the in vivo detection of blood glucose. The PA signal underwent preprocessing before being displayed on the Yokogawa DLM2034 Mixed Signal Oscilloscope (MSO). The MSO captured the averaged signal frame of the PA signal along with other essential metrics. The extracted features from the PA signal were fed as the inputs to the kernel-based ridge regression algorithm for the detection of blood glucose. The Raspberry Pi4 model b^38^ board was used in order for implementing machine learning algorithms. To further enable real-time blood glucose monitoring, we utilized the ThingSpeak Cloud platform^39,40^ and Message Queuing Telemetry Transport (MQTT) protocol^41^ to secure the data transmission using Transport Layer Security (TLS).

### Experimental analysis

#### Hardware setup for in vivo testing of glucose detection

**Figure 2:**
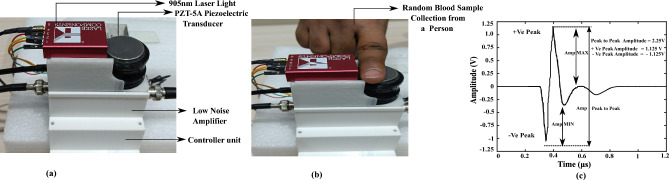
Experimental set up and data acquisition (**a**) The experimental setup of the Photoacoustic Spectroscopy device. (**b**) Photoacoustic Signal measurement is taken from a person of age 30 years. (**c**) Photoacoustic Signal generated from the device with different signal features.

A total of 105 random blood sample readings were collected from our device. Before taking the reading, each volunteer underwent physical measurements of their weight and height to calculate their BMI, and also measured other biological parameters like SpO2, Blood pressure, and Age. The study was conducted with the approval of the ethical committee of SRM University-AP, India, and all subjects were informed about the scientific use of their data. During the study, an experienced medical team utilized the Accu-Chek Performa II, (Roche Diagnostics GmbH, Germany) and Accu-Chek Softclix lancing device for the random glucose measurement, maintains the standards of ISO 15197:2013, while our equipment was specifically configured to record the 1024 time frame of averaged PA signal. Masimo Rad-5v Handheld Pulse Oximeter is utilized for the measurement of SpO2 and it maintains the standards of ISO 80601-2-61:2017. The blood pressure was measured with the Omron M3 Comfort Upper Arm Blood Pressure Monitor and it follows the standards of ISO 81060-2:2018. In order to maintain accuracies, all of the devices adhere to ISO standards for measurements. The experimental setup of the device is shown in Fig. 2a. The process of data acquisition from a person shown in Fig. 2b and the features like Peak-to-peak amplitude, RMS values, Positive, and Negative Peaks of the PA signal were captured from the device for a person and it was depicted in Fig. 2c. The feature values are varied from person to person with the change in the blood glucose values. Among all the features we observed peak-to-peak amplitude of a PA signal varied mostly with the glucose values.

#### Preprocessing

**Figure 3:**
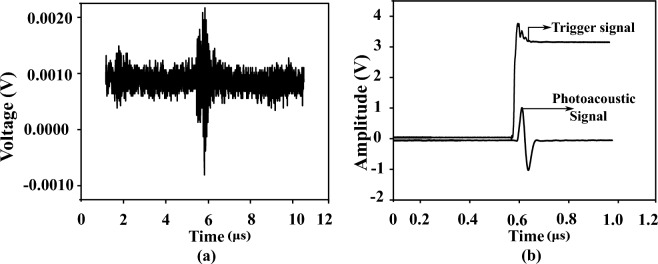
PA signal processing (**a**). Acquired noisy signal before preprocessing. (**b**). Representing the amplified processed signal.

The electrical response of the transducer is low and usually measured in microvolts ($\mu$V). A pre-amplifier and amplification circuits are necessary to improve this weak signal. The piezoelectric transducer is susceptible to random noise in its operating environment due to its fragile output and it was shown in Fig. 3a. Additional forms of distortions include noise from various biological sources and electromagnetic interference from power lines. Hence, amplification of the noise-corrupted signal must be performed by considering the Signal-to-Noise Ratio (SNR). To reduce the noise and enhance the quality of the signal a Low-Noise Amplifier (LNA) at the front end is necessary for optimal signal acquisition. The Analog Modules LNA with model number 351A-2-50-NI is chosen due to its low drift and low noise characteristics. The amplifier operated in the non-inverting mode with a variable gain of 40−60 dB and a bandwidth of −3 dB. The amplification adds minimal noise to the signal and the processed PA signal is shown in Fig. 3b.

#### Feature extraction

**Figure 4:**
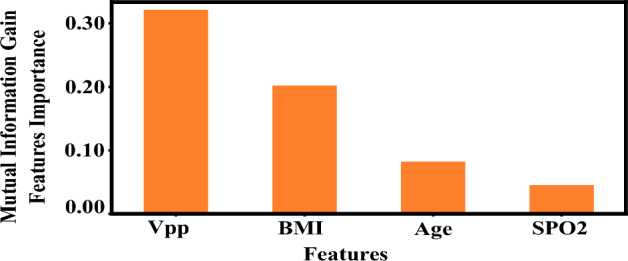
Representation of the feature importance from the Mutual Information Gain algorithm in the corresponding order.

The amount of glucose produced corresponds to the PA pressure signal received by the sample. The calibration relies on the amplitude and area-based features of the recorded PA signal. As a result, it’s essential to extract the relevant details from the PA signal generated. In the PA signal, we have the parameters like Peak to Peak Amplitude values V$_{pp}$, Root Mean Square values (RMS), Positive and Negative peak amplitude values, maximum peak (Amp max), and minimum peak (Amp min). The V$_{pp}$ value is considered in the algorithm as it varied depending on a person’s individual glucose levels. Furthermore, we extracted the biological features of the individuals like BMI, Age, and SpO2 for better augmentation. To trace out the importance of the features from our dataset here Mutual Information Gain (MI)^42,43^ algorithm has been implemented for the feature selection and the resulting important features of the MI algorithms are shown in Fig. 4.

#### Calibration for glucose estimation

To estimate glucose levels, the PA measurements obtained from a sample are processed, and n number of signal features, denoted as X j,i are computed. These features are used for the creation of the feature vector, and the estimation of glucose P$_g$ is shown in Eq. (3).

3 $$\begin{aligned} P_g = [x_{j,1}, x_{j,2},...x_{j,n}]^T \end{aligned}$$

By utilizing signal features, x$_{j,i}$, extracted from the PA response of a sample p$_g$ and the estimation of the sample’s glucose concentration, est$_{cg}$, can be obtained through a calibration process. The calibration function should accurately capture the relationship between the signal features and changes in glucose concentration in order to provide precise estimates of the sample’s glucose concentration. The signal features from P$_g$ are used to create a feature vector $\varphi (P_{g})$ for a d dimensional calibration function given in Eq. (4)

4 $$\begin{aligned} \varphi (P_{g}) = \left\{ \prod _{i=1}^{n}P_{j,i}^{b_{i}}:\sum _{i=1}^{n}b_{i}\le d \right\} \varepsilon \mathbb {R}^{\left( n+d\right) } \end{aligned}$$

To estimate the glucose concentration est$_{cg}$ a calibration model takes the input features from the PA measurement. The model produces a set of coefficient values $\theta$. The obtained features are applied to a new PA measurement x$_i$, the estimated glucose can be written as Eq. (5)

5 $$\begin{aligned} est_{C_{g}} = \theta ^T\varphi (P_i) \end{aligned}$$

Comparisons between the estimated glucose concentrations derived from a set of m measurements and the reference glucose concentrations have been made and to compute the estimation value error, J($\theta$) can be written in Eq. (6)

6 $$\begin{aligned} J(\theta ) = \sum _{i=1}^{m}(\theta ^{T}\varphi (x_{i})-ref_ {C_{g}})^2 \end{aligned}$$

The J ($\theta$) represents the mean square error value. To address the computational and storage challenges associated with an increasing number of signal features and the dimension of the calibration function, kernels are employed. Kernels offer a solution by eliminating the need for the explicit creation and storage of the complete input feature vector. The kernel-based ridge regression^44^ can be expressed in the following form

7 $$\begin{aligned} g(\varphi (x_j)) = \left\langle w,\phi (x)) \right\rangle = \left( \sum _{i=1}^{l}\alpha _i\phi (x_i),\phi (x))\right) \end{aligned}$$

Now apply the gram matrix to the Eq. (7) then the equation can be converted into the following form

8 $$\begin{aligned} g(\varphi (x_j))= & {} \sum _{i=1}^{l}\alpha _i \left\langle \phi (x_i),\phi (x)\right\rangle \end{aligned}$$

9 $$\begin{aligned} g(z)= & {} K( x_i, x) \end{aligned}$$

Putting the value of g(z) in Eq. (8) and by applying kernel trick representation theorem^45–48^ the set of the linear combination of training data is represented in the following form.

10 $$\begin{aligned} g(\varphi (x_j))= \sum _{i=1}^{m}\alpha _i k(x_i,x) \end{aligned}$$

k represents the kernel function and we are implementing the ridge regression with the polynomial kernel of degree 3, the polynomial kernel is represented as in Eq. (11)

11 $$\begin{aligned} k_{poly}(x_i,x_j)= (1+(x_i)^Tx_j)^d . \end{aligned}$$

m is the size of the training set and it contains the input feature vector with a set of features like BMI, Age, and SpO2, calculated from each measurement. The BMI as one of the features from the feature vector has been implemented in the proposed algorithm. The utilization of larger feature sets in the prediction function enhances its expressive power but also increases the risk of overfitting the training data. To mitigate this issue, the optimization process takes into account both the estimation error and the coefficient weights. Introducing a regularization parameter, $\lambda$ allows for fine-tuning the trade-off between the two factors. By carefully adjusting these parameters, the prediction function can strike a clear balance between model complexity and generalization performance. It effectively avoids the overfitting and improves the overall accuracy, reliability of the predictions which can be shown in the following Eq. (12)

12 $$\begin{aligned} J(\theta ) = min\left\| k \alpha -ref C_{g} \right\| ^2_2 + \lambda \left\| f \right\| ^2_k. \end{aligned}$$

k is the training data of the kernel matrix and $ref_{cg}$ is the reference glucose concentration and the set of reference measurements taken from the individuals

$$\begin{aligned} K(l,m) = K_{l,m}= K(P_l,P_m) \end{aligned}$$

$\left\| f \right\| ^2_k$ is the Kernel Hilbert Space norm of the objective function and it is given as

13 $$\begin{aligned} \left\| f \right\| ^2_k = \alpha ^T k\alpha . \end{aligned}$$

The Eq. (12) can be written as

14 $$\begin{aligned} J(\theta )= min\left\| k \alpha -ref _{cg} \right\| ^2_2 + \lambda \alpha ^T k\alpha . \end{aligned}$$

Computing the gradient to the function Eq. (14) the $\alpha ^*$ is obtained as

15 $$\begin{aligned} \alpha ^* = ( k+ \lambda I)^-1 ref_{cg}. \end{aligned}$$

Using Eqs. (15) and (10) the estimation of glucose can be written as

16 $$\begin{aligned} est _{cg} = ref _{cg}^T(K+\lambda I)^-1 K_i \end{aligned}$$

The selection of the kernel degree and regularization parameter is crucial for accurate prediction and avoiding overfitting in polynomial kernel ridge regression. The kernel degree controls the complexity of the model, and the regularization parameter balances the trade-off between fitting the training data and generalization. Values for these parameters are methodically explored, and validation data is used for assessment to establish the best combination. The prediction accuracy is evaluated by quantitatively comparing the estimated glucose concentration $est_{cg}$ to the reference glucose measurements $ref_{cg}$. Metrics such as Root Mean Square Error (RMSE), Mean Absolute Difference (MAD), and Mean Absolute Relative Difference (MARD) are considered to precisely assess the accuracy of the prediction of glucose. These variables provide crucial details about the model’s overall effectiveness in estimating the desired outputs by assessing the degree of agreement between the model’s predictions and actual values.

The systematical exploration of various parameter combinations and calculation of the RMSE values for each combination using different kernel operations are reported in Table 1. The amplitude value from the PA signal combined BMI value of a person produced the best results, where BMI is a scientifically relevant characteristic that has a more pronounced impact on glucose concentration^30,49,50^. The trace out of the above combination Table 2 is implemented for different types of kernel operations with BMI and peak-to-peak amplitude value of the PA signal. We have calculated the metrics for each kernel and traced out ridge regression polynomial kernel which shows the better result for all the metrics. A dataset of 105 samples is used to train the model, and the dataset is split into training and testing portions in an 80:20 ratio. The anticipated outcomes of the model were further examined graphically by the Clarke Error Grid Analysis (CEGA) and Bland Altman Plot (BAP) which demonstrates the predictable accuracy and dependability of the model.

**Table 1:** Identification of RMSE values with various physical parameters with PA signal voltage value.

RMSE values (mg/dl)					
Specifications	Polynomial	Laplacian	Linear	RBF	Additive Chi
Only with Vpp	11.34	11.13	14.62	11.22	156.50
Vpp, SpO2	11.06	11.20	11.77	11.88	266.35
Vpp, BMI, SpO2	11.73	13.31	11.59	16.57	10.97
Vpp, BMI, AGE	14.31	43.02	15.08	67.01	13.19
Vpp, BMI, Age, SpO2	14.61	37.15	13.23	70.24	13.04
Vpp, BMI	10.94	14.31	12.81	16.29	11.08

**Table 2:** Statistical parametric values of the different kernels in a ridge regression with PA signal voltage and BMI value.

Kernel calibration model	Signal features	RMSE (mg/dl)	MAD (mg/dl)	MARD (%)
Linear	Vpp, BMI	12.81986	13.24607	11.60143
Laplacian	Vpp, BMI	14.31098	10.44692	9.684433
RBF	Vpp, BMI	16.29039	12.73354	12.0672
Additive Chi	Vpp, BMI	11.08715	11.15355	9.745183
Polynomial	Vpp, BMI	10.94	10.15365	8.861604

#### Integration of IoT to the proposed system

The Raspberry Pi 4 model b board was used for our prototype device. The Raspberry Pi is configured with a 64-bit Raspbian OS in the dedicated memory card of 16 GB. We have found that the tensor flow 2.6 version is compatible for the execution of machine learning algorithms on the Raspberry pi board. The Kernel based ridge regression is implemented on the Raspberry pi model b^38^ board. The obtained glucose values from the algorithm along with the reference glucose values are transmitted to the ThingSpeak cloud platform^39,40^. The Message Que Telemetry Transport (MQTT)^41^ protocol has been used to secure the data transmission to the ThingSpeak cloud using Transport Layer Security (TLS). This combination enables data integrity, authentication, and encryption to provide a secure communication relationship. Furthermore, an Application Programming Interface (API) key generated on the platform is used to authenticate with the ThingSpeak cloud. The API key serves as a secure credential that enables the application to safely access and send data to the ThingSpeak cloud. The process of secure communication includes server authentication, creating a TLS-encrypted connection, and sending data across this secure channel. ThingSpeak account is set up to enforce secure connections and limit access to the devices with working API credentials.

## Results and discussion

The estimated values from the polynomial kernel-based ridge regression are analyzed in CEGA^51,52^ and BAP^53,54^. The CEGA divides the glucose measurement space into five zones based on the reference glucose value and the predicted glucose value. The zones represent different degrees of clinical significance in terms of the impact on patient management. The values plotted in Zone-A are the most accurate predictions of glucose values and they fall within an acceptable range of the reference values. Predictions in this zone are considered clinically safe, as they would likely lead to appropriate treatment decisions. The values which are in Zone-B deviate from the reference values but without significant clinical consequences. Finally, the values which are plotted in Zones C to E are clinically not acceptable. By visually assessing the distribution of glucose values in the different zones, healthcare professionals can quickly evaluate the clinical relevance and accuracy of glucose predictions. Similarly, BAP is to used visualize the agreement between the methods and allows for the identification of any systematic bias or variable bias. The plot typically includes a horizontal axis representing the mean difference between the two methods and the vertical axis represents the difference between two values. The limits of agreement indicates the range within which most differences between the two methods fall. About 95% of the variations between the two methods of measuring data can be found within the range indicated by the 95% limits of agreement.

**Figure 5:**
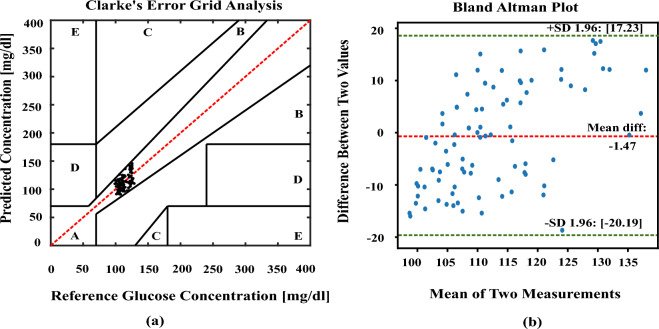
Error plots analyze the differences between actual and estimated glucose concentrations using polynomial kernel-based ridge regression for all data samples (105 samples) (**a**). CEGA shows the visual representation of the alignment between the real and predicted glucose values. (**b**) BAP shows the average bias and variability between two assessment methodologies.

**Figure 6:**
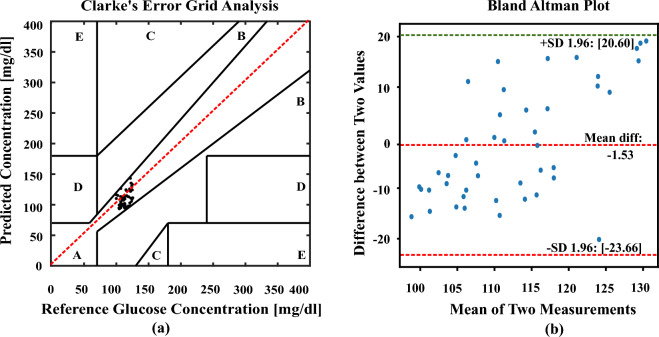
Error plots analyze the differences between actual and estimated glucose concentrations using polynomial kernel-based ridge regression for the normal BMI cluster data samples (52 pairs of data) (**a**). CEGA shows the visual representation of the alignment between the real and predicted glucose values. (**b**) BAP shows the average bias and variability between two assessment methodologies.

**Figure 7:**
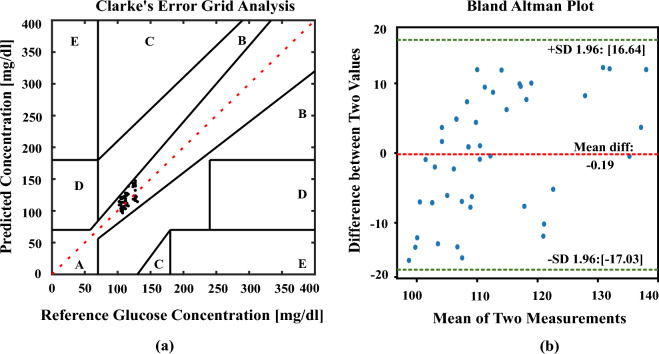
Error plots analyze the differences between actual and estimated glucose concentrations using polynomial kernel-based ridge regression for the obese and overweight BMI cluster data samples (53 pairs of data) (**a**) CEGA shows the visual representation of the alignment between the real and predicted glucose values. (**b**) BAP shows the average bias and variability between two assessment methodologies.

**Figure 8:**
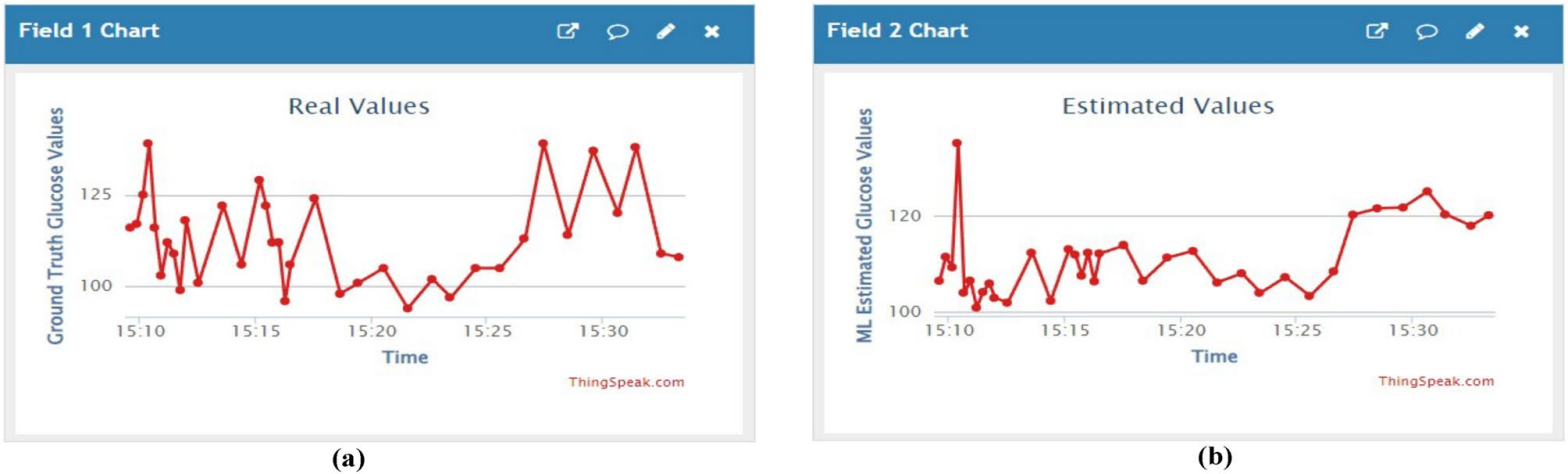
Real values and Estimated values of the glucose from the model are visualized on the ThingSpeak Cloud platform. (**a**) Ground Truth Values, (**b**) Estimated Values obtained from the model.

Here in our work, we have collected 105 random average blood samples from our prototype device and the accuracy of the result is presented using Fig. 5a CEGA and Fig. 5b BAP plots which shows 100% of our estimated glucose values are projected in the Zone-A of CEGA and also falls in between the upper and lower threshold values of BAP. The respective RMSE, MAD, and MARD for this category are calculated as 10.94 (mg/dl), 10.13 (mg/dl), 8.86% and the mean difference, upper and lower limit of agreement values of BAP are -1.47, 17.23,-20.19 respectively. To further analyze the samples, the data is converted

into two clusters based on the values of BMI. In the first cluster a normal BMI value which is less than 25 is considered and the data size of the cluster is 52 pairs of random blood sample readings. In the other cluster the BMI greater than 25 is considered as obese and overweight persons and the size of the data is 53 pairs. In both clusters, we utilized the PA signal amplitude value and the BMI value of a person as variables in the algorithm. The training and testing of the data are divided into 80:20 ratio. The graphical plot of the Fig. 6a CEGA and Fig. 6b BAP shows the accuracy of normal BMI cluster. The statistical parameters values of RMSE, MAD, and MARD are obtained as 11.80 (mg/dl), 10.35 (mg/dl), and 9.08%, and the mean difference, upper

and lower limit of agreement values of BAP are -1.53, 20.60, -23.66 respectively. Finally, the plots in Fig. 7a CEGA and Fig. 7b BAP validate the second cluster. The values of RMSE, MAD, and MARD are obtained as 11.32 (mg/dl), 9.92 (mg/dl), 8.67% and the mean difference, upper and limit of agreement values of BAP are -0.19, 16.64, -17.03 respectively. The obese person’s estimated values are nearer to the ground truth values which are depicted by our algorithm. We have found that all the estimated values fall in the Zone-A of CEGA and in between the line of agreement values of BAP in all the clusters. It is identified that the impact of BMI and the PA signal amplitude value plays a crucial role for noninvasive in vivo detection of random blood glucose from the CEGA, and BAP. Finally, the IoT technique has been implemented using the Thing Speak Cloud Platform for the real-time monitoring of the data. For every 15 seconds, the data is sent to the cloud of each person and the visual plots of the cloud platform have shown in Fig. 8.

### Comparison analysis

In comparison to the existing methods for blood glucose measurement noninvasively using PAS both in vivo and in vitro, our proposed technique outperforms in terms of MAD, MARD, and RMSE readings. The comparative study is shown in Table 3. Through our in vivo operations, we have created a sophisticated method that uses the PA signal’s peak-to-peak amplitude value to offer precise and accurate results using BMI. A significant number of the estimated samples are found to be concentrated in Zone A of the Clarke Error Grid Analysis (CEGA). Additionally, the MARD values obtained from the algorithm supported for clinical acceptance. However, more samples have to be tested with the same algorithm to determine consistent outcomes before contemplating clinical use. These findings are further supported and detailed in Table 4, providing conclusive evidence of the efficiency and usefulness of our strategy in obtaining the estimation of precise results.

**Table 3:** Comparison analysis of the statistical parameters for the glucose estimation by previously reported works using Photoacoustic Spectroscopy.

Ref no	Parameters	MAD values (mg/dl)	MARD values (%)	RMSE values (mg/dl)	Implementation approach	Regression algorithm	No of data samples
Pai et al. 2015^55^	Peak to peak voltage values	15.27	11.78	N.A	In vitro	Linear	30
Pai et al. 2017^19^	Peak to peak voltage values	N.A	8.84	N.A	In vitro	Polynomial	24
Pai et al. 2018^21^	Dual frequency voltage values	5.23	2.07	7.64	In vitro	Gaussian	30
Sim et al. 2018^20^	Microscopic spatial information of finger at non secreting points through image	8.27	9.94	N.A	In vivo	PLSR	23 pairs
	Microscopic spatial information of finger at secreting points through image	11.98	14.67	N.A			
Long et al. 2021^23^	Imaging on human tissue	N.A	N.A	24.24	In vitro	PLSR	45
	Imaging simulated data (10dB)	N.A	N.A	15.69			
	Imaging simulated data (30dB)	N.A	N.A	13.15			
The proposed method (i) All collected samples	Vpp, BMI Values	10.153	8.861	10.941	In vivo	Ridge with Polynomial Kernel	105 pairs of random blood samples
(ii) Normal BMI values		10.352	9.080	11.795			
(iii) Obese and overweight BMI values		9.921	8.866	11.316

**Table 4:** Comparison of CEGA result with previously reported works.

Reference	Technique	Zone A(%)	Zones A &B(%)	Zones C-E(%)	MARD (%)	Method
^19^	Peak to peak voltage values using photoacoustic spectroscopy	92.86	100.00	0.00	8.84	In vitro
^21^	Dual frequency peak to peak voltage values using photoacoustic spectroscopy	100.00	100.00	0.00	2.07	In vitro
^56^	Diffuse reflectance	87.50	95.80	4.20	–	In vivo
^7^	Raman spectroscopy	86.66	–	–	–	In vitro
^12^	Mid Infra red spectroscopy	84.00	–	–	–	In vivo
^10^	Optical coherence tomography	83.00	99.00	1.00	11.50	In vivo
^55^	Photoacoustic spectroscopy	82.65	100.00	0.00	11.78	In vitro
^57^	Photoacoustic spectroscopy	66.50	94.60	5.40	–	In vivo & in vitro
^20^	Photoacoustic spectroscopy	70	100.00	0.00	11.98	In vivo
Proposed method	Photoacoustic spectroscopy with Vpp, BMI value	100.00	100.00	0.00	8.86	In vivo

## Conclusions

The proposed IoT based noninvasive in vivo glucose detection approach is based on PAS with random blood sample collection. In this work, we traced out that the voltage measurements from the PA signal alone might not be adequate for calibration using the Kernel-based Polynomial ridge regression. Therefore we integrated different biological parameters of the body in the algorithm with the PA signal amplitude values. We tried different possible combinations of the samples and executed the algorithm with the best kernel result. We calculated the estimated values of glucose from all the clusters with the combination of PA signal voltage value and BMI in the polynomial-based kernel ridge regression. Finally, we achieved a better outcome in terms of MAD, MARD, and RMSE values for noninvasive in vivo detection of random blood glucose compared to the prior state of the arts. Moreover, our estimated values are lies in the Zone-A of CEGA in all the clusters and in the range of upper and lower threshold values of BAP. Finally, for real-time data monitoring, we deploy the ML regression algorithm at the device level and identify the estimated values on the Thing Speak cloud platform. The execution time of our algorithmic method using an Intel Core i5-8500T CPU @2.10 GHz processor is found 26.412 ms, representing the result of the calculation time produced by our experimental setup.

## Study subjects

The sensor model was trained and evaluated on a generic population made up of healthy individuals in the age group of 23- 65 years included in our study. The study protocol confirms the ethical guidelines of the 1964 Declaration of Helsinki, and the Indian Council of Medical Research (ICMR) 2000, which was approved by the medical officer and ethical committee at SRM University-AP India. Using the Accu-Chek Performa II (Roche Diagnostics GmbH, Germany) and Accu-Chek Softclix lancing devices, the trained medical staff of SRM University-AP took 105 random blood glucose readings from different subjects. Each subject gave their informed consent for the scientific use of data.

## Data Availability

The Data is confidential and it cannot be disclosed in an open-source platform at the present movement for this paper. However, it will be made available on reasonable requests to the corresponding author.
